# Human amnion-derived mesenchymal stem cells alleviate lung injury induced by white smoke inhalation in rats

**DOI:** 10.1186/s13287-018-0856-7

**Published:** 2018-04-12

**Authors:** Pei Cui, Haiming Xin, Yongming Yao, Shichu Xiao, Feng Zhu, Zhenyu Gong, Zhiping Tang, Qiu Zhan, Wei Qin, Yanhua Lai, Xiaohui Li, Yalin Tong, Zhaofan Xia

**Affiliations:** 1 Research Laboratory of Burns and Trauma, the 181st Hospital of Chinese PLA, Guilin, 541002 People’s Republic of China; 2 Department of Burns, Plastic and Wound repair surgery, the 181st Hospital of Chinese PLA, Guilin, 541002 People’s Republic of China; 3 Trauma Research Center, First Hospital Affiliated to the Chinese PLA General Hospital, Beijing, 100048 People’s Republic of China; 4 Department of Burn surgery, Changhai Hospital, Naval Military Medical University, Shanghai, 200433 China

**Keywords:** White smoke inhalation, Human amnion-derived mesenchymal stem cells, Lung injury, Cell-based therapy

## Abstract

**Background:**

White smoke inhalation (WSI) is an uncommon but potentially deadly cause of acute lung injury and acute respiratory distress syndrome for which no effective pharmaceutical treatment has been developed. This study aimed to determine the protective effects of human amnion-derived mesenchymal stem cells (hAMSCs) against WSI-induced lung injury in rats.

**Methods:**

hAMSCs were injected into rats via the tail vein 4 h after WSI. At 1, 3, 7, 14, and 28 days after cell injection, hAMSCs labeled with PKH26 in lung, heart, liver, and kidney tissues were observed by fluorescence microscopy. The lung injury score was determined by hematoxylin and eosin staining. Lung fibrosis was assessed by Masson’s trichrome staining. The computed tomography (CT) score was assessed by CT scanning. The wet/dry weight ratio was calculated. The levels of interleukin (IL)-1β, IL-6, and IL-10 were determined by enzyme-linked immunosorbent assays. The expression of surfactant protein (SP)-A, SP-C, and SP-D was measured by Western blotting.

**Results:**

The injected hAMSCs were primarily distributed in the lung tissues in WSI-induced rats. Compared with the model and phosphate-buffered saline (PBS) group, hAMSC treatment led to reduced lung injury, lung fibrosis, CT score, and inflammation levels in WSI-induced mice. hAMSC treatment also resulted in increased cell retention in the lung, partial pressure of oxygen (PaO_2_), and PaO_2_/fraction of inspired oxygen (FiO_2_) levels, and pulmonary SP-A, SP-C, and SP-D expression compared with that in the model and PBS group.

**Conclusions:**

hAMSCs are a potential cell-based therapy for WSI-induced lung injury.

## Background

Smoke pots and smoke bombs, which are widely used in fire drills or retreats, can produce white smoke. Compared to the smoke generated by the combustion of tobacco or wood dust, white smoke not only contains carbon tetrachloride, carbon monoxide, and carbon dioxide, but is also mixed with other highly corrosive, irritating, and toxic substances that include tetrachloroethylene, zinc chloride, hexachloroethane, and oxides (zinc oxide, aluminum oxide, iron oxide, etc.), which increase the damage caused by white smoke inhalation (WSI) [1–3]. Accidental exposure to high concentrations of white smoke, particularly in a confined space, can lead to inhalation injury, acute respiratory distress syndrome, pulmonary fibrosis, and even death [4, 5]. Many studies have reported that lung injury can be caused by a single or several components in white smoke, and confirmed that zinc chloride and hexachloroethane are important substances as inducers of lung injury [6–8]. Currently, no clinical treatments are available. Therefore, it is necessary to develop effective therapeutic methods to treat lung injury induced by white smoke.

Mesenchymal stem cells (MSCs) are multipotent cells that can differentiate into a variety of lineages. MSCs have the potential to repair injured tissues by secreting growth factors and anti-inflammatory molecules [9]. MSC therapy may be a promising therapeutic strategy for treatment of white smoke-induced acute respiratory distress syndrome. It has been reported that MSCs may recover lung fibroblast function from cigarette smoke-induced damage [10, 11]. Human amnion-derived MSCs (hAMSCs) can be collected from the amnion, which is generally discarded as medical waste. Thus, there are fewer ethical issues limiting hAMSC collection [12]. Previous studies demonstrated that hAMSCs have anti-inflammatory effects in dextran sulfate sodium-induced severe colitis and carbon tetrachloride-induced liver fibrosis [13, 14] and that hAMSCs alleviate lung injury induced by lipopolysaccharide and ischemia and reperfusion [15, 16].

However, the therapeutic efficacy of hAMSCs against lung injury induced by WSI has not been examined. In this study, we investigated whether tail vein injection of hAMSCs could improve WSI-induced lung injury in rats.

## Methods

### Dissociation and culture of primary hAMSCs

All pregnant women or their relatives provided written informed consent for sample collection. The study was conducted according to principles of the Helsinki Declaration of 1975 as revised in 1983 and was approved by the Ethics Committee of No. 181 Hospital of the People’s Liberation Army. Primary hAMSCs were dissociated from fresh placenta specimens of full-term pregnancies delivered by cesarean section. Briefly, the amniotic membrane was shaken with the same volume of 0.25% trypsin-0.02% EDTA-disodium salt solution at 200 rpm for 50 min at 37 °C, and the supernatant was discarded. This step was repeated twice. Next, the amniotic membrane was cut into pieces and shaken with the same volume of 0.2 mg/mL type IV collagenase containing 0.075 mg/mL DNase I (Worthington, Lakewood, NJ, USA) for 2 h. The solution was filtered through a 200-mesh sieve (bore diameter = 0.074 mm) and centrifuged at 1000 g at 4 °C for 5 min. The cells were washed twice with phosphate-buffered saline (PBS) and resuspended in Dulbecco’s modified Eagle’s medium (DMEM) supplemented with 10% fetal bovine serum (FBS; Gibco, Grand Island, NY, USA). Then, 5 mL of the cell suspension cells (1 × 10^6^ cells/mL) in a 25-cm^2^ flask were maintained at 37 °C in a humidified atmosphere of 95% air and 5% CO_2_. After 48 h, the culture medium and nonadherent cells were discarded and fresh medium was added. Cell growth was observed under an inverted phase contrast microscope (Olympus, Tokyo, Japan). When growth reached 80% confluence, cells were digested and cultured in DMEM supplemented with 10% FBS. Third-generation hAMSCs were digested and collected, and the hAMSC surface markers were measured using a Human MSC Analysis Kit (BD Biosciences, San Diego, CA, USA) and a FACSCalibur flow cytometer (BD Biosciences) equipped with Cell Quest software (BD Biosciences).

### Establishment of the WSI model and treatment

**Fig. 1:**
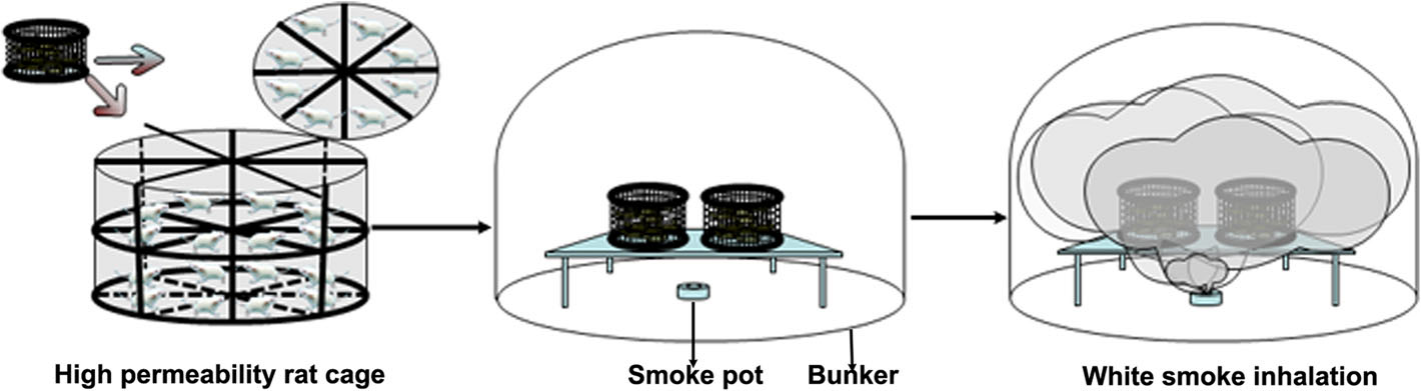
Schematic representation of the establishment of the WSI model

All rats were purchased from the animal center of Guilin Medical College. Rat protocols were approved by the ethics committee of the No. 181 Hospital of the People’s Liberation Army and disposal methods followed animal ethical standards. Rats were allowed free access to water and food and were housed at 20 to 25 °C in a room that was continuously ventilated with 12 h of illumination and 12 h of darkness. The experimental steps to establish the WSI model are presented in Fig. 1. The rats were placed in a high-permeability rat cage with an isolation network to physically separate the rats and to filter the impact of white smoke. The high-permeability rat cage was placed in the center of a 2-m^2^ homemade laboratory apparatus used for smoke injury research (Patent No.: 201720002689.0). A smoke pot was located 60 cm below the rat cage. The smoke pot was ignited, burned, and gradually produced smoke. The WSI time was calculated from the beginning of smoke generation. After WSI for 5 min, the rats were fed under normal conditions. At 4 h post-WSI, rats were randomly divided into the model, PBS, and hAMSC treatment groups. Rats in the model group were not injected with hAMSCs. Rats in the PBS group each received a tail vein injection of 200 μL PBS. Each rat in the hAMSC treatment group received a tail vein injection of 200 μL PBS containing 1 × 10^6^ hAMSCs. Normal rats that were not treated (*n* = 6) comprised the control group.

### Computed tomography (CT) evaluation

At 1, 3, 7, 14, and 28 days post-treatment, rats were fixed to a foam board in the supine position under general anesthesia induced using chloral hydrate. The rat breast was scanned with a 64-slice GE LightSpeed VCT (GE Healthcare, Little Chalfont, UK) at an 80 kV tube voltage, 150 mA tube current, and medium bowtie filter (0.625 mm half-value layer). CT scans were scored according to the radiologist’s score method as previously described [17]. Briefly, each image slice was divided into four quadrants (i.e., right and left ventral, and right and left dorsal) and each quadrant was assigned a numeric score of 0 (normal), 1 (interstitial markings), 2 (ground-glass opacification), or 3 (consolidation). The worst possible score was given to each quadrant, that is the presence of a small area of consolidation in a quadrant yielded a score of 3 even if the remainder of the quadrant was not consolidated. This score was added across all quadrants and slices to determine the total radiologist’s score for the entire scan. A thoracic radiologist blinded to the group assignments and sampling time point evaluated each scan score according to the total radiologist’s score. The CT baseline value was 52.83 ± 2.14. Theoretically, the CT score is 0. However, individual image artifacts produced a score between 0 and 52.83.

### Arterial blood gas levels

One milliliter of noncoagulated blood was collected from the abdominal aorta of rats. Each sample was analyzed to determine the partial pressure of oxygen (PaO_2_), partial pressure of carbon dioxide (PaCO_2_), and the ratio of PaO_2_ to the fraction of inspired oxygen (PaO_2_/FiO_2_) using a blood gas analyzer (Radiometer Medical, Copenhagen, Denmark).

### Assessment of hAMSC localization

hAMSCs were labeled with PKH26 red fluorescent dye before tail vein injection. PKH26 labeling has no effect on the hAMSC phenotype or the behavior of the cells in vitro or in vivo, and is a safe and effective way to label hAMSCs [18, 19]. After treatment, the rats were sacrificed and the lung, heart, liver, and kidney tissues were collected and frozen at −80 °C. The frozen tissues were continuously cut into 4-μm slices that were observed by fluorescence microscopy (magnification × 100, Olympus, Tokyo, Japan).

### Wet/dry weight ratio

After anesthetic overdose and exsanguination (by severing the inferior vena cava and abdomen aorta), lung lobes were weighed (wet weight), placed in an oven, and weighed daily until the weight was unchanged (dry weight). The wet/dry weight ratio was calculated from the initial and final values.

### Assessment of pathological changes

Pathological changes in lung tissues were visualized by hematoxylin and eosin (H&E) staining. The diaphragmatic leaves of the right lung of rats from each group were fixed with 4% formalin, washed, dehydrated, embedded in paraffin, and cut into 4-μm sections. H&E staining was performed using a kit according to the manufacturers’ instructions (Solarbio, Beijing, China). Semi-quantitative scoring of bleeding, edema, and inflammation of lung tissues was performed by optical microscopy examination (magnification × 200, Olympus). The degree of lung injury was graded as described previously [20]. The pathological score was graded as follows: a score of 0 indicated no alveolitis; a score of 1+ indicated mild alveolitis with thickening of the alveolar septum by a mononuclear cell infiltrate, with involvement limited to focal, pleural-based lesions occupying < 20% of the lung and with good preservation of the alveolar architecture; a score of 2+ indicated moderate alveolitis with more widespread alveolitis involving 20 to 50% of the lung, although still predominantly pleural based; finally, a score of 3+ indicated severe alveolitis with diffuse alveolitis involving more than 50% of the lung, with occasional consolidation of air spaces by the intra-alveolar mononuclear cells and some hemorrhagic areas within the interstitium and/or alveolus. Tissue sections were also analyzed by Masson’s trichrome staining. Ten random fields on a section from each rat were photographed and blue-stained areas were calculated from the entire lung cross-sectional area (%, × 20) with a digital image analyzer (WinROOF; Mitani Co., Fukui, Japan) to evaluate the degree of lung fibrosis. The degree of lung fibrosis was graded as follows: a score of 0 indicated no evidence of fibrosis; a score of 1+ indicated mild lung fibrosis with focal regions of fibrosis involving < 20% of the lung, with the fibrosis involving the pleura and the interstitium of the subpleural parenchyma with some distortion of alveolar architecture; a score of 2+ indicated moderate fibrosis, which was evident as more extensive fibrosis involving 20 to 50% of the lung and fibrotic regions that mostly extend inward from the pleura and still focal; finally, a score of 3+ indicated severe fibrosis, which was evident as widespread fibrosis involving more than 50% of the lung and the presence of confluent lesions with extensive derangement of parenchymal architecture, including cystic air spaces lined by cuboidal epithelium.

### Measurement of inflammatory cytokines

The right lung was separated and the right main bronchus was ligated. Next, 1 mL of ice saline was injected from the end of the trachea and washed by lavage three times. A 2.6-mL sample of bronchoalveolar lavage fluid was collected and centrifuged at 350 g for 10 min at 4 °C. Transforming growth factor (TGF)-β1, tumor necrosis factor (TNF)-α, interleukin (IL)-6, and IL-10 levels in the supernatant of bronchoalveolar lavage fluid were measured with enzyme-linked immunosorbent assay (ELISA) kits (Multi Science, Hangzhou, Zhejiang, China).

### Western blotting

Western blot analysis was conducted to analyze protein expression in the lung tissue. Briefly, protein lysis buffer was added to minced lung tissue and the tissue was homogenized. The samples were centrifuged at 10,000 g at 4 °C. Protein concentration in the supernatant was quantified using a BCA protein quantification kit (Tiangen, Beijing, China). Aliquots containing 50 μg total proteins were used for 8% sodium dodecyl sulfate-polyacrylamide gel electrophoresis and the resolved proteins were transferred to a nitrocellulose membrane (BioTrace Medical, Menlo Park, CA, USA). The membranes were incubated with 1:1000 dilutions of antibodies against pulmonary surfactant protein (SP)-A, SP-C, SP-D, and glyceraldehyde-3-phosphate dehydrogenase (GAPDH) (all from Cell Signaling Technology, Danvers, MA, USA) at 4 °C on a rotating shaker overnight. The membranes were subsequently washed with Tris buffered saline-Tween (TBST) three times for 5 min each time, followed by incubation with horseradish peroxidase-labeled goat anti-rabbit IgG (H + L; Beijing ZSGB Bio, Beijing, China, 1:5000 dilution) for 1 h at 25 °C on a horizontal shaker. The membranes were washed with TBST twice for 5 min each time, followed by chemiluminescence detection. The protein band areas were quantified using Image Lab 6.0 software (Bio-Rad, Hercules, CA, USA). The GAPDH antibody was used as an internal reference.

### Statistical analyses

Statistical analyses were performed using SPSS v19.0 software (SPSS, Inc., Chicago, IL, USA). All data are expressed as the mean ± standard deviation (SD). The data were analyzed by one-way analysis of variance followed by post-hoc tests of the least significant difference for multiple pairwise comparisons. *P* < 0.05 was considered statistically significant.

## Results

### hAMSC morphology and verification of phenotype verification

**Fig. 2:**
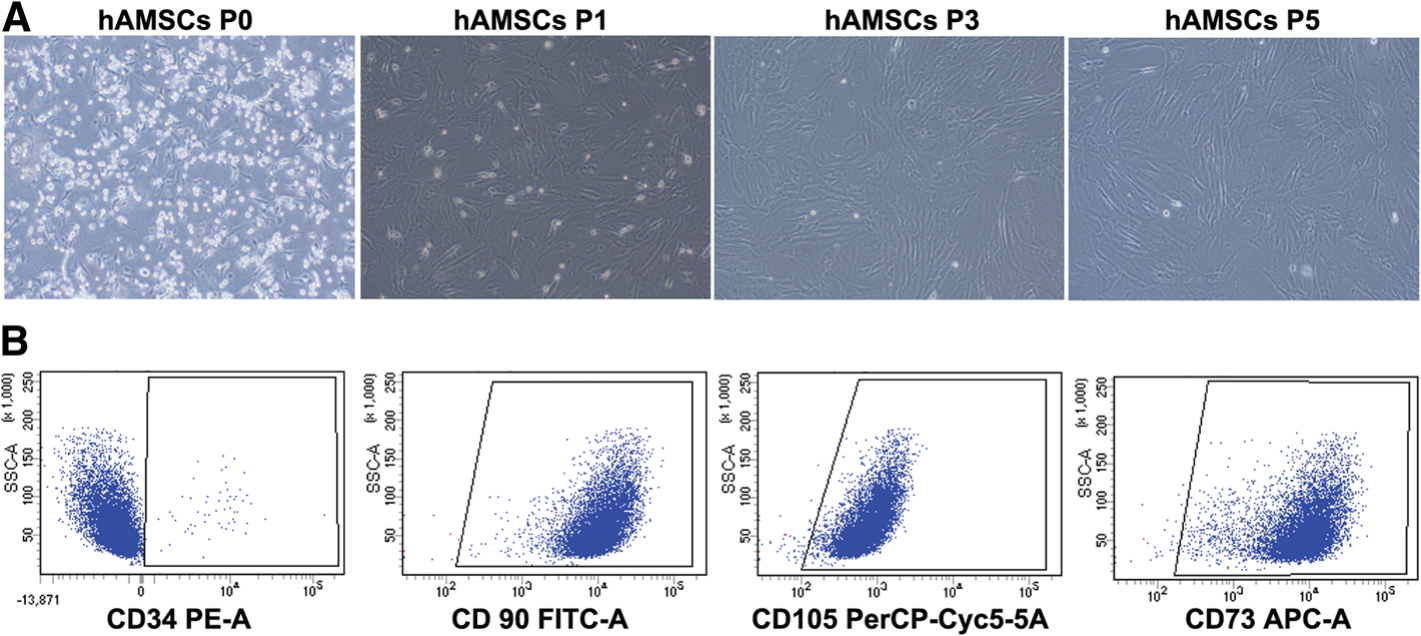
Validation of human amnion-derived mesenchymal stem cell (hAMSC) properties. **a** hAMSCs were characterized at passage (P)0, P1, P3, and P5 by morphology. **b** Purity was checked by flow cytometric analysis of CD34, CD90, CD105, and CD73 expression in hAMSCs. The panel shows the results of gene expression analysis of positive (CD90, CD105, and CD73) and negative (CD34) MSC markers

hAMSCs displayed MSC characteristics including adhesion and a spindle-shaped and flat morphology (Fig. 2a). hAMSCs were negative for CD34 and positive for CD105, CD90, and CD73 mesenchymal markers (Fig. 2b).

### Distribution of hAMSCs in WSI-induced rats

**Fig. 3:**
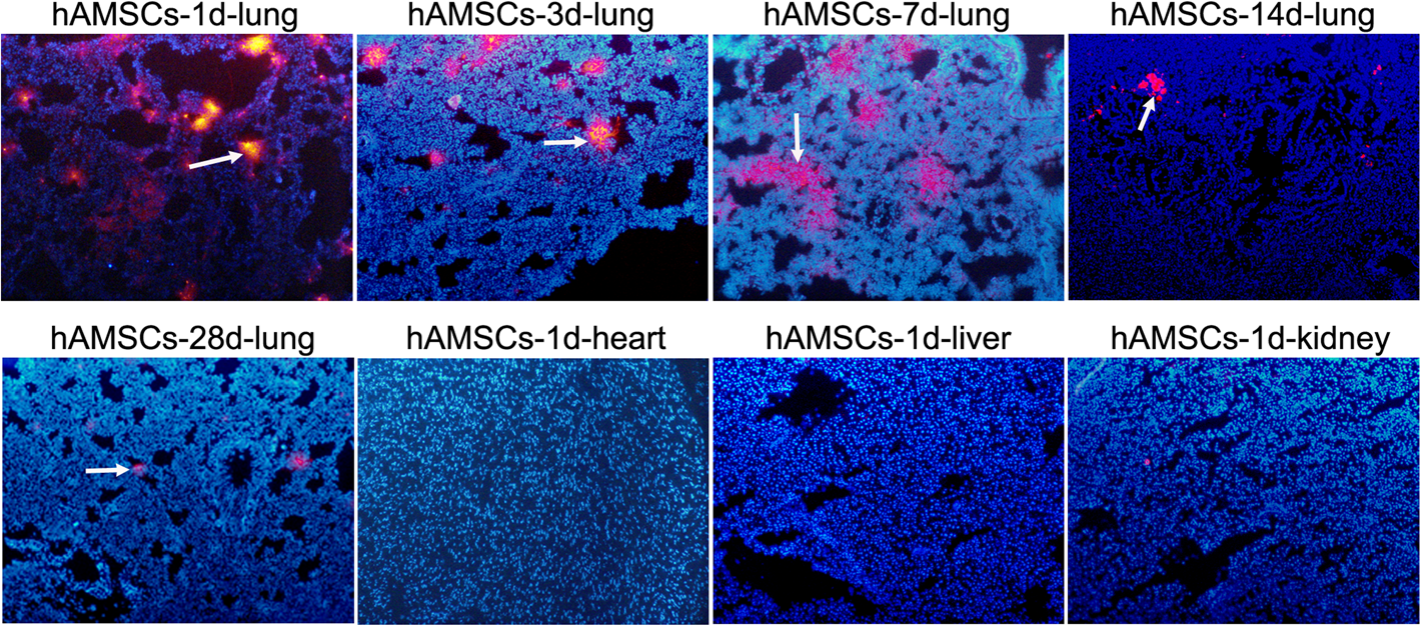
Distribution of human amnion-derived mesenchymal stem cells (hAMSCs) in the lung (at 1, 3, 7, 14, and 28 days after hAMSC treatment), heart, liver, and kidney tissues observed by fluorescence microscopy

hAMSCs labeled with PKH26 in the lung, heart, liver, and kidney tissues were observed using fluorescence microscopy (Fig. 3). hAMSCs were clearly observed in the lung tissues but were gradually reduced at 1, 3, 7, 14, and 28 days after treatment with hAMSCs. Additionally, nearly no hAMSCs were detected in the heart, liver, and kidney tissues. The results suggest that hAMSCs mainly migrated to the lung tissue in WSI-induced rats.

### CT score increased by WSI is reduced by hAMSC treatment

**Fig. 4:**
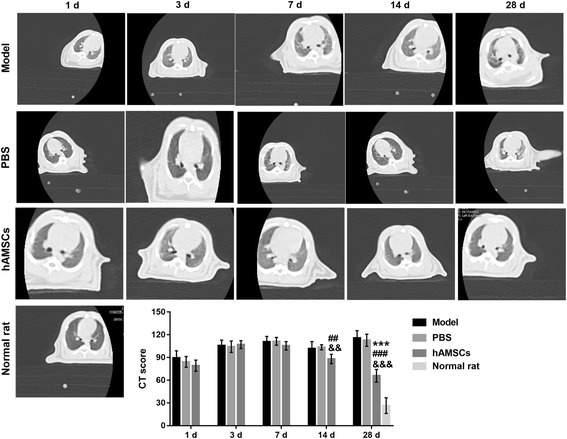
The computed tomography (CT) score was measured at 1, 3, 7, 14, and 28 days after treatment by CT scanning. CT score data are presented as the mean ± SD. ***P < 0.001, vs. at 1 day after human amnion-derived mesenchymal stem cell (hAMSC) treatment. ^##^*P* < 0.01, ^###^*P* < 0.001, vs. model group; ^&&^*P* < 0.01, ^&&&^*P* < 0.001, vs. phosphate-buffered saline (PBS) group

CT scans of WSI-induced rats after treatment revealed varying increases of lung marking, ground-glass opacities, and lung consolidation in the model and PBS groups at 1, 3, 7, 14, and 28 days after treatment (Fig. 4). In the hAMSC treatment group, the CT score was increased at 3 and 7 days after treatment followed by reductions at 14 and 28 days after treatment. The pathological score was significantly reduced at 28 days after hAMSC treatment compared with that at 1 day after hAMSC treatment (*P* < 0.05). Compared with the model group and PBS group, the CT score in the hAMSC group was significantly decreased at 28 days post-hAMSC treatment (*P* < 0.001). However, the CT score did not decrease to the normal value after hAMSC treatment at 28 days.

### Wet/dry weight ratio in WSI-induced rats is reduced by hAMSC treatment

**Fig. 5:**
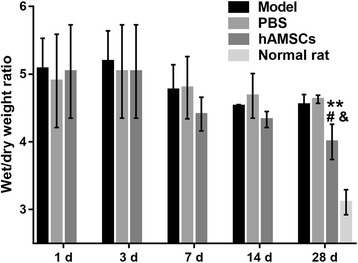
Wet/dry weight ratio was measured at 1, 3, 7, 14, and 28 days after treatment. Data for the wet/dry weight ratio are presented as the mean ± SD. **P < 0.01, vs. at 1 day after human amnion-derived mesenchymal stem cell (hAMSC) treatment. ^#^*P* < 0.05, vs. model group; ^&^*P* < 0.05, vs. phosphate-buffered saline (PBS) group

At 1, 3, 7, 14, and 28 days after treatment, the wet/dry weight ratio was measured (Fig. 5). The wet/dry weight ratio gradually decreased after hAMSC treatment and was significantly reduced at 28 days after hAMSC treatment compared with that at 1 day after hAMSC treatment. Additionally, the wet/dry weight ratio was significantly decreased at 28 days after hAMSC treatment compared with that in the model group and PBS group at the same time point (*P* < 0.05). However, the wet/dry weight ratio did not decrease to the normal value after hAMSC treatment at 28 days.

### Pathological score increased by WSI is reduced by hAMSC treatment

**Fig. 6:**
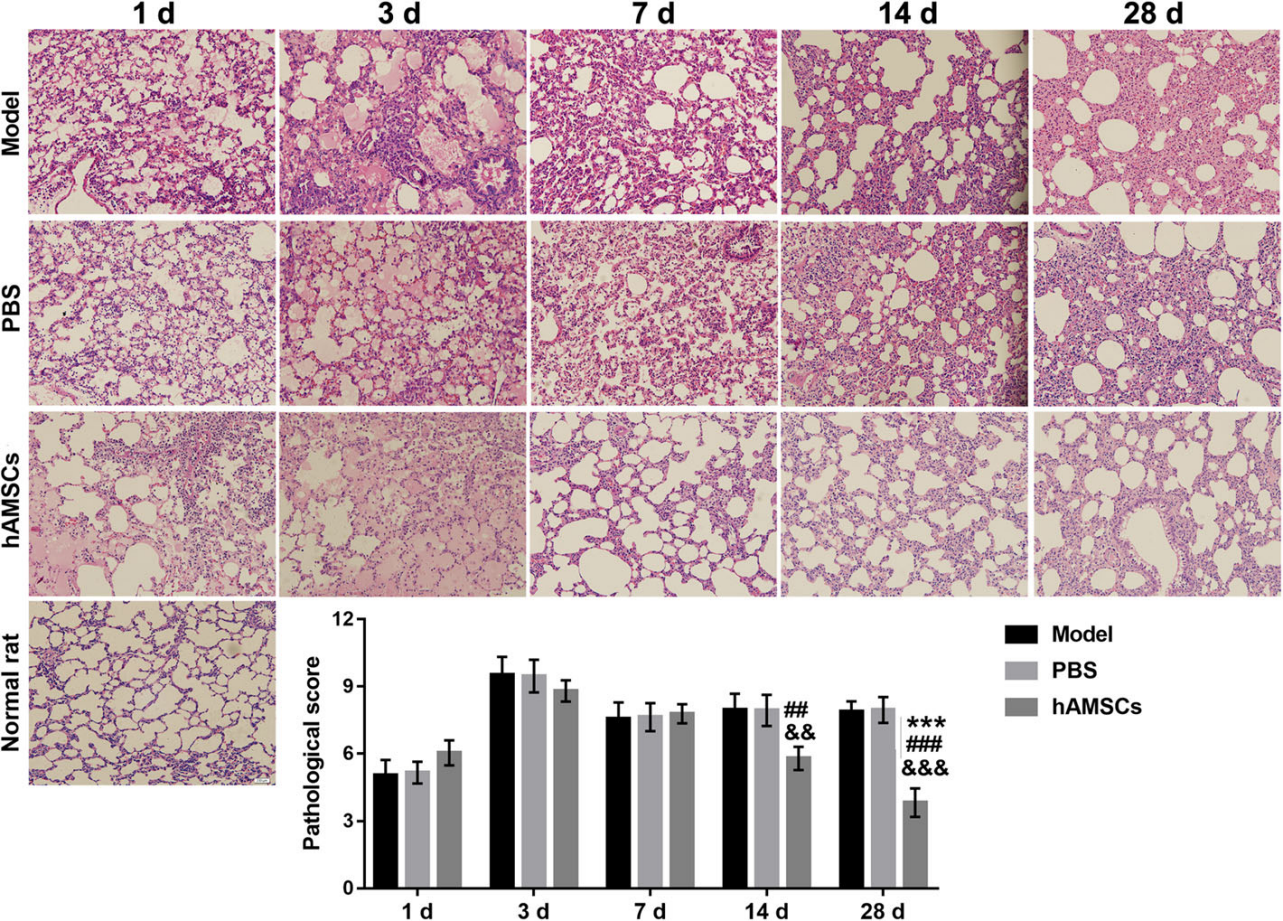
Histopathological features were tested by H&E staining at 1, 3, 7, 14, and 28 days after treatment in WSI-induced rats and the pathological score was measured. Data for pathological score are presented as the mean ± SD. Pathological score in the normal rats is 0. ***P < 0.01, vs. at 1 day after human amnion-derived mesenchymal stem cell (hAMSC) treatment; ^##^*P* < 0.01, ^###^*P* < 0.001, vs. model group; ^&&^*P* < 0.01, ^&&&^*P* < 0.001, vs. phosphate-buffered saline (PBS) group

Histopathological features of lung tissue were evaluated by H&E staining. The lung tissue of WSI-induced rats in the model, PBS, and hAMSC treatment groups exhibited histopathological features of lung injury including congestion of airway mucosa, edema, and bleeding, neutrophil infiltration, alveolar wall thickening, and alveolar wall collapse (Fig. 6). In the hAMSC treatment group, the pathological score was increased at 3 days after treatment followed by reductions at 7, 14, and 28 days after treatment. The pathological score was significantly reduced after hAMSC treatment at 14 and 28 days compared with after hAMSC treatment at 1 day (*P* < 0.05). Additionally, the pathological scores after hAMSC treatment at 14 and 28 days were significantly decreased compared with those in the model group and PBS group at the same time point (*P* < 0.01). However, the pathological score did not decrease to the normal value after hAMSC treatment at 28 days.

### Lung fibrosis grade increased by WSI is reduced by hAMSC treatment

**Fig. 7:**
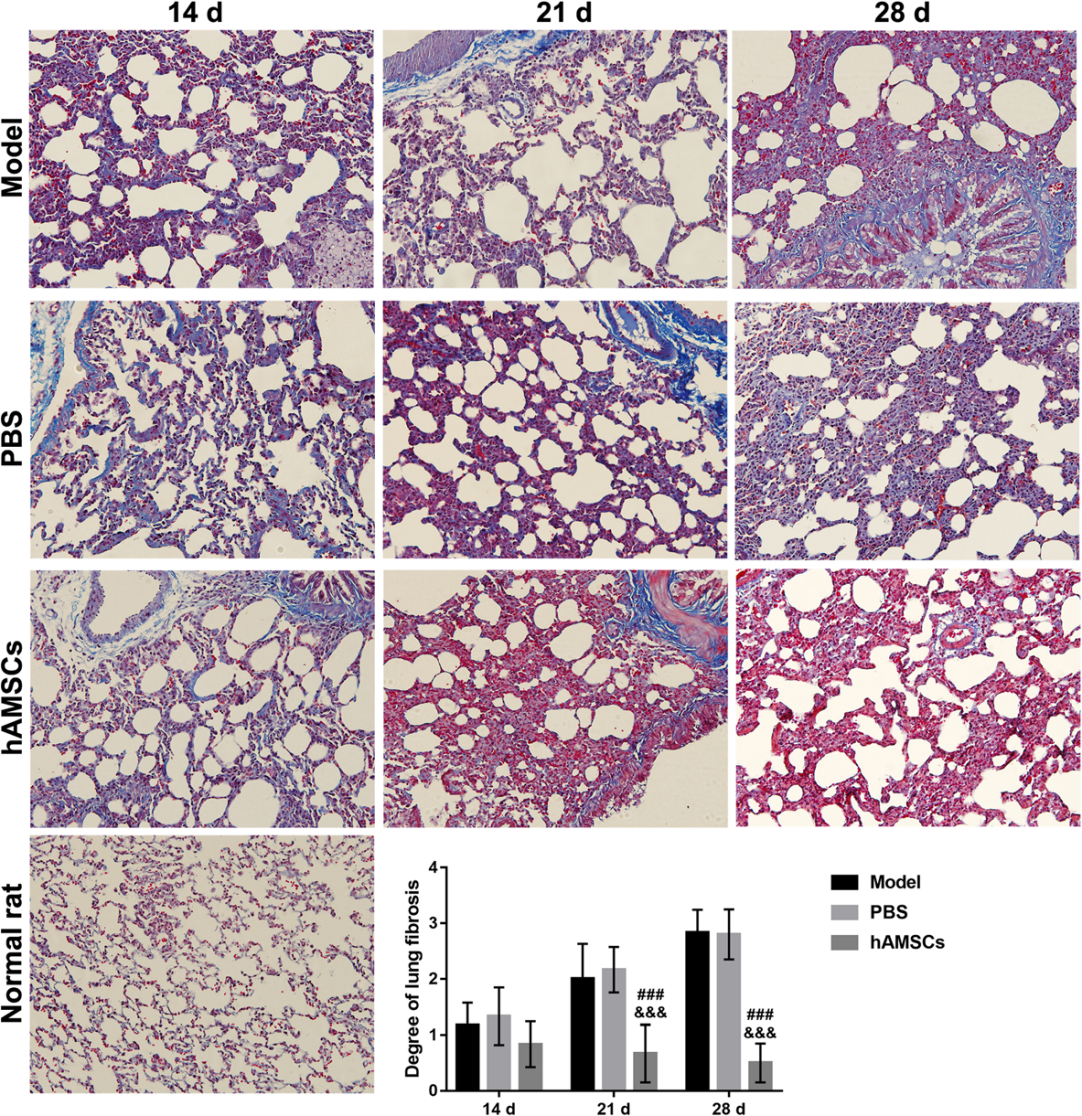
Lung fibrosis was measured at 14, 21, and 28 days after treatment by Masson trichrome staining. The degree of lung fibrosis is presented as the mean ± SD. The degree of lung fibrosis in the normal rat is 0. ^###^*P* < 0.001, vs. model group; ^&&&^*P* < 0.001, phosphate-buffered saline (PBS) group. hAMSC, human amnion-derived mesenchymal stem cell

Lung fibrosis was measured at 14, 21, and 28 days after treatment by Masson trichrome staining (Fig. 7). In the hAMSC treatment group, lung fibrosis was reduced at 14, 21, and 28 days after treatment. Lung fibrosis in the hAMSC treatment group at 14 and 28 days was significantly decreased compared with that in the model group and PBS group at the same time point (*P* < 0.001). However, the lung fibrosis grade did not decrease to the normal value after hAMSC treatment at 28 days.

### hAMSC treatment alleviates the changes in arterial blood gases induced by WSI

**Fig. 8:**
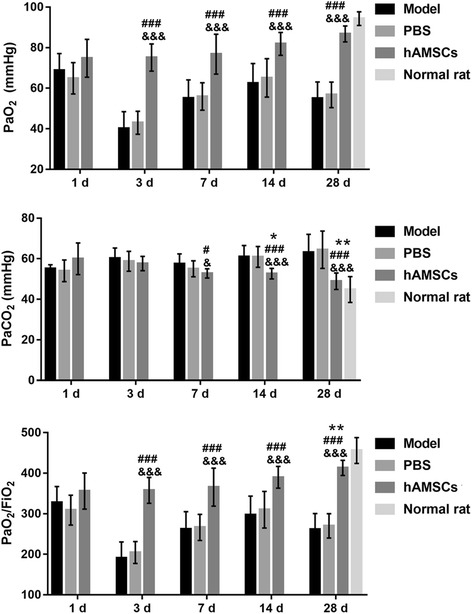
Arterial blood gas was measured at 1, 3, 7, 14, and 28 days after treatment. Data are presented as the mean ± SD. *P < 0.05, **P < 0.01, vs. at 1 day after human amnion-derived mesenchymal stem cell (hAMSC) treatment; ^#^*P* < 0.05, ^###^*P* < 0.001, vs. model group; ^&^*P* < 0.05, ^&&&^*P* < 0.001, phosphate-buffered saline (PBS) group. FiO_2_, fraction of inspired oxygen; PaCO_2_, partial pressure of carbon dioxide; PaO_2_, partial pressure of oxygen

Figure 8 depicts the levels of PaO_2_, PaCO_2_, and PaO_2_/FiO_2_ in WSI-induced rats after treatment. The levels of PaO_2_ and PaO_2_/FiO_2_ in the hAMSC treatment group gradually increased, while the level of PaCO_2_ gradually decreased at 1, 3, 7, 14, and 28 days after treatment. The levels of PaO_2_ and PaO_2_/FiO_2_ were significantly enhanced after hAMSC treatment at 28 days compared with that after hAMSC treatment at 1 day (*P* < 0.01), while the levels of PaO_2_ and PaO_2_/FiO_2_ were significantly reduced after hAMSC treatment at 14 and 28 days compared with that after hAMSC treatment at 1 day (*P* < 0.05). The levels of PaO_2_ and PaO2/FiO_2_ were significantly increased (*P* < 0.001), while the level of PaCO_2_ was significantly inhibited (*P* < 0.01), at 7, 14, and 28 days after hAMSC treatment compared with that in the model group and PBS group at the same time points (*P* < 0.01). However, the levels of PaO_2_, PaCO_2_, and PaO_2_/FiO_2_ did not recover to the normal values after hAMSC treatment at 28 days.

### hAMSC treatment increases the expression of SP-A, SP-C, and SP-D decreased by WSI

**Fig. 9:**
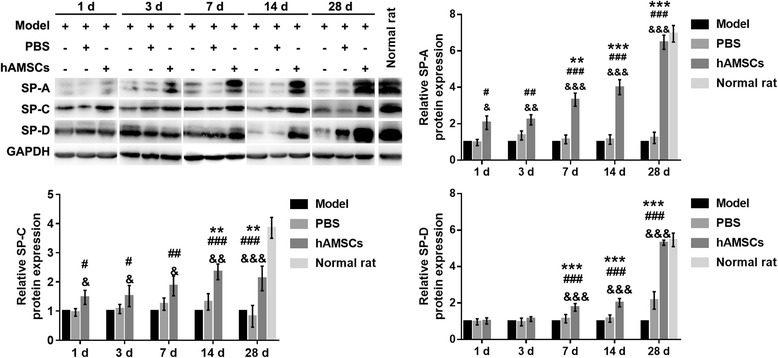
Expression of surfactant protein (SP)-A, SP-C, and SP-D was evaluated by Western blotting at 1, 3, 7, 14, and 28 days after treatment. Protein expression data are presented as the mean ± SD. **P < 0.01, ***P < 0.001, vs. at 1 day after human amnion-derived mesenchymal stem cell (hAMSC) treatment; ^#^*P* < 0.05, ^##^*P* < 0.01, ^###^*P* < 0.001, vs. model group; ^&^*P* < 0.05, ^&&^*P* < 0.01, ^&&&^*P* < 0.001, vs. phosphate-buffered saline (PBS) group. GAPDH, glyceraldehyde-3-phosphate dehydrogenase

The expressions of SP-A, SP-C, and SP-D were measured by Western blotting (Fig. 9). The expressions of SP-A, SP-C, and SP-D gradually increased at 1, 3, 7, 14, and 28 days after hAMSC treatment. The expressions of SP-A, SP-C, and SP-D were significantly increased at 7, 14, and 28 days after hAMSC treatment compared with that after hAMSC treatment at 1 day (*P* < 0.01). The expressions of SP-A, SP-C, and SP-D were increased after hAMSC treatment compared with that in the model group and PBS group at the same time point. However, the SP-A, SP-C, and SP-D expressions did not increase to the normal values after hAMSC treatment at 28 days.

### hAMSC treatment reduced the levels of inflammatory cytokines increased by WSI

**Fig. 10:**
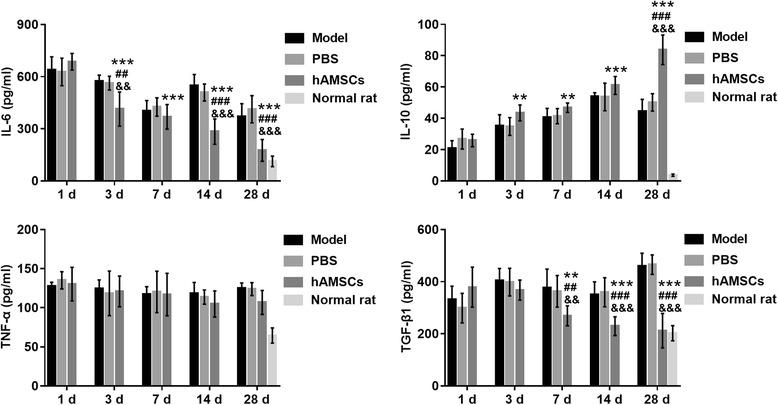
Inflammatory cytokine levels were measured by ELISA at 1, 3, 7, 14, and 28 days after treatment. Data are presented as the mean ± SD. **P < 0.01, ***P < 0.001, vs. at 1 day after human amnion-derived mesenchymal stem cell (hAMSC) treatment; ^##^*P* < 0.01, ^###^*P* < 0.001, vs. model group; ^&&^*P* < 0.01, ^&&&^*P* < 0.001, vs. phosphate-buffered saline (PBS) group. IL, interleukin; TGF, transforming growth factor; TNF, tumor necrosis factor

The levels of inflammatory cytokines were measured by ELISA (Fig. 10). The levels of IL-6, TNF-α, and TGF-β1 were gradually decreased, while the level of IL-10 was gradually increased at 1, 3, 7, 14, and 28 days after hAMSC treatment. The levels of IL-6 and TGF-β1 were significantly reduced at 7, 14, and 28 days after hAMSC treatment compared with those after hAMSC treatment at 1 day (*P* < 0.01), while the level of IL-10 was significantly enhanced after hAMSC treatment at 3, 7, 14, and 28 days compared with that at 1 day after hAMSC treatment (*P* < 0.05). However, the level of TNF-α was not significantly reduced at 3, 7, 14, and 28 days after hAMSC treatment compared with that at 1 day after hAMSC treatment. The levels of IL-6, TNF-α, and TGF-β1 were decreased, while the level of IL-10 was increased (*P* < 0.01) after hAMSC treatment at 3, 7, 14, and 28 days compared with those in the model group and PBS group at the same time point, but the level of TNF-α was not significantly reduced. However, the levels of inflammatory cytokines did not recover to the normal values after hAMSC treatment at 28 days.

## Discussion

Clinical studies revealed that patients severely affected by exposure to white smoke display significant pathological characteristics that include pulmonary edema, bleeding, inflammatory infiltration, diffuse alveolar damage, and pulmonary fibrosis [21–23]. CT scanning results have shown similar characteristics as pulmonary vascular lesions in adult respiratory distress syndrome caused by inhalation of zinc chloride smoke [24]. In this study, we found that rats induced by WSI exhibited congestion of airway mucosa, edema and bleeding, neutrophil infiltration, alveolar edema and diffuse bleeding, alveolar wall thickening, and alveolar wall collapse. Lung fibrosis, lung marking, ground-glass opacities, and lung consolidation were increased to different degrees after WSI in rats, similar to the pathological characteristics of WSI patients. hAMSCs can proliferate in lung tissues and improve lung injury induced by lipopolysaccharide and ischemia and reperfusion [15, 25]. Similar to the findings of previous studies, we found that hAMSCs introduced by tail vein injection become primarily distributed in the lung tissues in WSI-induced rats, and that hAMSC treatment reduced lung injury and lung fibrosis, and decreased the CT score increased by WSI according to the results of CT scanning, H&E staining, and Masson trichrome staining. The wet/dry weight ratio and degree of pulmonary edema were increased after smoke inhalation in an ovine model [26]. In this study, we found that the wet/dry weight ratio was gradually decreased after hAMSC treatment compared with that in the WSI model, similar to the findings from H&E-stained lungs, suggesting that the degree of pulmonary edema was decreased after hAMSC treatment. These results suggest that hAMSC treatment improves lung injury induced by WSI, however, lung injury did not recover to the normal values after hAMSC treatment at 28 days. Additionally, we found that edema is no longer evident at 14 and 28 days according to H&E results in the model and PBS groups; rather, an increase in fibrosis is observed at these time points in the model and PBS groups, with possible reasons being that white smoke could persist in the airways for a long time and inflame lung tissue due to the tiny particles in the smoke, resulting in infiltrated collagen fibers in most areas of the lung. Although edema was no longer apparent in the model and PBS groups, it was still present in the lung tissue at these time points and the reasons for this still need further verification.

As pattern-recognition molecules of the collectin family of C-type lectins, SP-A, SP-C, and SP-D play important roles in mediating pulmonary immune defense [27]. The changes in SP-A, SP-C, and SP-D caused by cigarette smoking contribute to the development of lung diseases [28]. Previous studies also showed that smoke inhalation injury frequently induces severe hypoxemia and increases the risk of acute respiratory distress syndrome [29]. Our results showed that hAMSC treatment increased SP-A, SP-C, and SP-D expressions, increased PaO_2_ and PaO_2_/FiO_2_ levels, and decreased PaCO_2_ levels compared with those in the WSI model. These results suggest that hAMSC treatment can improve the respiratory and immune functions inhibited by WSI. However, the respiratory and immune functions did not recover to the normal values after hAMSC treatment at 28 days. Furthermore, we found that PaO_2_ and the PaO_2_/FiO_2_ ratio were significantly increased at all time points starting from day 3 in the hAMSC-treated group compared with the PBS and model groups, but the levels of lung injury were similar in all three groups at day 3 and day 7. Possible reasons are as follows: PaO_2_ and the PaO_2_/FiO_2_ ratio do not correspond exactly to the degree of lung injury since the body automatically begins respiratory compensation under acute hypoxia and, therefore, PaO_2_ and the PaO_2_/FiO_2_ ratio do not decrease. However, when the body is heavily damaged, and the compensatory respiration range is exceeded, PaO_2_ and the PaO_2_/FiO_2_ ratio cannot increase. In our study, PaO_2_ and the PaO_2_/FiO_2_ ratio decreased significantly on day 3 in the PBS and model groups compared with the hAMSC-treated group because, in the PBS and model groups, each body was heavily damaged, and the compensatory respiration range was exceeded, resulting in decrease in PaO_2_ and PaO_2_/FiO_2_ ratio. With the recovery of the body, the body entered the compensatory respiration range, and PaO_2_ and the PaO_2_/FiO_2_ ratio increased on day 7 in the PBS and model groups. However, PaO_2_ and the PaO_2_/FiO_2_ ratio exhibited no significant change with hAMSC treatment. Additionally, pathological results could reflect the state of lung injury. We observed that on days 3 and 7, the degrees of lung injury in all three groups were similar because the therapeutic effects of hAMSCs on lung injury take some time to manifest.

Inflammatory cytokines, such as TGF-β1, TNF-α, IL-1β, and IL-10, act as tracheobronchial markers of lung injury in smoke inhalation victims [29]. TGF-β1, TNF-α, and IL-1β inhibit lung injury repair and promote lung cell apoptosis and fibrosis [30, 31], the levels of which are increased by cigarette smoke [32]. Reduced pulmonary inflammation contributes to remodeling of lung injury [33, 34]. IL-10, an anti-inflammatory cytokine, reportedly inhibits cigarette smoke-induced pulmonary neutrophilic inflammation and TNF-α expression in mice [35]. In this study, we found that hAMSC treatment decreased the levels of IL-6, TNF-α, and TGF-β1, but increased the level of IL-10 compared with those in the WSI model. These results suggest that hAMSC treatment improves the levels of inflammatory cytokines changed by WSI. However, the levels of inflammatory cytokines did not recover to the normal values after hAMSC treatment at 28 days.

## Conclusions

In conclusion, hAMSC treatment can improve lung injury and respiratory and immune functions and inhibit the levels of inflammatory cytokines induced by WSI. These results implicate hAMSCs as a potential cell-based therapy for WSI-induced lung injury. However, the mechanism underlying hAMSC treatment for WSI-induced lung injury requires further investigation.

## Abbreviations

CT: Computed tomography
DMEM: Dulbecco’s modified Eagle’s medium
ELISA: Enzyme-linked immunosorbent assay
FBS: Fetal bovine serum
FiO_2_: Fraction of inspired oxygen
GAPDH: Glyceraldehyde-3-phosphate dehydrogenase
H&E: Hematoxylin and eosin
hAMSC: Human amnion-derived mesenchymal stem cell
IL: Interleukin
MSC: Mesenchymal stem cell
PaCO_2_: Partial pressure of carbon dioxide
PaO_2_: Partial pressure of oxygen
PBS: Phosphate-buffered saline
SD: Standard deviation
SP: Surfactant protein
TBST: Tris buffered saline-Tween
TGF: Transforming growth factor
TNF: Tumor necrosis factor
WSI: White smoke inhalation
